# The proteoglycan decorin does not influence adiposity, glucose tolerance, or aerobic exercise capacity in mice

**DOI:** 10.14814/phy2.70424

**Published:** 2025-07-04

**Authors:** Casey L. Egan, Atanaska Doncheva, Martin Pal, Sarah L. Fox, Knut Tomas Dalen, Martin Whitham, Mark A. Febbraio, Marit Hjorth

**Affiliations:** ^1^ Drug Discovery Biology, Monash Institute of Pharmaceutical Sciences Monash University Parkville Victoria Australia; ^2^ Garvan Institute of Medical Research Darlinghurst New South Wales Australia; ^3^ Department of Nutrition, Institute of Basic Medical Sciences, Faculty of Medicine University of Oslo Oslo Norway; ^4^ Faculty of Science and Health, School of Dentistry and Medical Sciences Charles Sturt University Wagga Wagga New South Wales Australia; ^5^ Cancer Epigenetic Biology and Therapeutics, Precision Medicine Theme Children's Cancer Institute Sydney New South Wales Australia; ^6^ School of Sport, Exercise and Rehabilitation Sciences University of Birmingham Edgbaston UK

**Keywords:** decorin, exercise, extracellular matrix, myokines, obesity

## Abstract

Physical activity is associated with improvements in insulin sensitivity and muscle function. The proteoglycan decorin is increased in skeletal muscle and plasma in response to exercise, but the biological implications are unknown. We investigated the effects of decorin deficiency on obesity, glucose tolerance, and exercise adaptation in C57BL/6J mice. Decorin deficiency did not influence adiposity, insulin‐ and glucose‐ tolerance, or energy metabolism in obese, high fat diet fed mice (*Dcn* ^−/−^ vs. *Dcn* ^+/+^). Decorin is abundant in the skeletal muscle extracellular matrix, thus we further compared the skeletal muscle of *Dcn* ^−/−^ and *Dcn* ^+/+^ littermates. There were no effects on muscle morphology or the expression of metabolic markers. *Dcn* ^−/−^ mice had normal exercise capacity measured as running distance on a treadmill. To study the effects of long‐term exercise, mice were housed with access to running wheels. Overall, there were no major differences in voluntary wheel running or skeletal muscle metabolic markers, but Dcn^−/−^ mice had a tendency for reduced running wheel activity compared to *Dcn* ^+/+^ mice. This was accompanied by a smaller exercise effect on metabolic markers in muscle Dcn^−/−^ mice. Our findings indicate that decorin does not have a major impact on glucose tolerance, metabolic adaptation, or aerobic exercise performance.

## Graphical abstract

Decorin deficiency does not have a major impact on glucose tolerance, metabolic adaptation, or aerobic exercise performance.

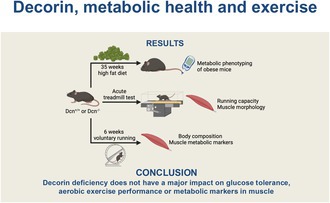

## 1 INTRODUCTION

Exercise improves insulin sensitivity and reduces the risk of type 2 diabetes (Colberg et al., 2016), but the underlying biological and molecular mechanisms are not fully understood. Long‐term physical activity is associated with reduced adiposity and low‐grade inflammation. Furthermore, exercise induces skeletal muscle adaptation, leading to improved muscle function and metabolism (Egan & Zierath, 2013). For instance, aerobic exercise increases mitochondrial density, oxidative capacity, and capillarization, while resistance exercise induces myofibrillar biosynthesis, leading to muscle hypertrophy.

Considerably less examined are the interactions of both obesity and physical activity on the extracellular matrix (ECM). Both appear to impact fundamental characteristics of the ECM (Hjorth et al., 2015; Martinez‐Huenchullan et al., 2017), such as its stiffness, remodeling, interaction with cells and mechanical signals, all of which are theorized to impact metabolic health (Huang & Greenspan, 2012). This highlights a potential additional avenue by which regular exercise may offer metabolic benefits via regulation and remodeling of the ECM by mechanisms not well understood.

We and others have identified decorin as an exercise‐responsive proteoglycan (Hjorth et al., 2024; Kanzleiter et al., 2014). We recently demonstrated that decorin is increased 1.55‐fold in skeletal muscle of mice after treadmill running, and by doing femoral, arteriovenous balance studies we demonstrated a net release of decorin from the exercising leg to the circulation in humans (Hjorth et al., 2024). This may indicate a role of decorin as an exercise‐induced myokine (Bekki et al., 2018; Kanzleiter et al., 2014; Langlois et al., 2025).

Decorin is abundant in the ECM of skeletal muscle and other tissues, where it may interact with multiple ECM proteins (Buraschi et al., 2019; Xie et al., 2022). It is involved in structural organization of collagen fibers, and deficiency leads to fragile skin (Danielson et al., 1997). Furthermore, decorin can influence cellular signaling by interacting with growth factors and cell surface receptors, including epidermal growth factor receptors (EGFR/ErbB1) (Santra et al., 2002), hepatocyte growth factor receptor (Goldoni et al., 2009), and Transforming growth factor beta (Yamaguchi et al., 1990). It has been demonstrated that decorin can inhibit myostatin and thereby indirectly influence proliferation and differentiation of muscle cells in vitro (Kishioka et al., 2008; Miura et al., 2006). A few studies have indicated a role for decorin in metabolically associated diseases; increased adipose tissue decorin has been associated with insulin resistance (Bolton et al., 2008), and decorin deficient mice gained more weight and had a lower glucose tolerance as compared to wild type mice on a high fat diet (Svärd et al., 2019). Importantly, the biological impact of exercise‐induced decorin is currently unknown.

In this study, we investigated the role of decorin in metabolism, exercise capacity, and skeletal muscle adaptation, hypothesizing that a lack of decorin will be associated with impaired metabolic function and subsequently exercise capacity.

## 2 MATERIALS AND METHODS

### 2.1 Animal experiments

Experiments on mice were approved by the Alfred Medical Research Education Precinct or Garvan Institute/St Vincent's Animal Ethics Committees, in accordance with the Australian code for the care and use of animals for scientific purposes. *Dcn* ^−/−^ mice were produced by the Mouse Engineering Garvan/ABR (MEGA) Facility using CRISPR/Cas9 gene targeting in mouse embryos following established molecular and animal husbandry techniques (Yang et al., 2014). Single guide RNAs (sgRNA) were produced based on target sites flanking Exon 3 of *Dcn* (GCCTAAGCCTACTAAATACT*TGG* and ACCTACAGTTAGATCAAAAC*AGG*, protospacer‐associated motif = PAM italicized and underlined). The two sgRNAs (15 ng/μL each) were microinjected into the nucleus and cytoplasm of C57BL/6J zygotes together with polyadenylated *S. pyogenes* Cas9 mRNA (30 ng/μL). Microinjected embryos were cultured overnight, and those that underwent cleavage were then introduced into pseudo‐pregnant foster mothers. A founder mouse was identified that carried a single 292 bp deletion in *Dcn* that removed Exon 3 (113 bp) plus 164 bp of 5′ and 16 bp of 3′ intronic sequences. The founder was backcrossed with syngeneic partners and then inter‐crossed to establish the *Dcn^−/−^* line on a pure C57BL/6J genetic background. Heterozygous males and females were bred to produce *Dcn* ^+/+^, *Dcn* ^*+/−*^, and *Dcn* ^*−/−*^ littermates for experiments. All genotyping was performed by the Garvan molecular genomics (GMG) facility. We used male and female mice for different experiments, which was primarily done to reduce the number of animals needed. Male mice were used for metabolic phenotyping in response to a high‐fat diet, as males tend to develop more insulin resistance. Muscle morphology was assessed in both male and female mice, but only males were used for acute exercise testing, while females were exposed to long‐term wheel running.

Mice were housed in a 12:12 h light:dark cycle, with access to food and water ad libitum. The standard chow diet (Specialty Feeds, Glen Forrest, Australia) consisted of 14.3 MJ/kg energy, with 76 E% carbohydrate, 5 E% fat, and 19 E% protein. To promote obesity, male *Dcn* ^+/+^ and *Dcn* ^−/−^ mice were fed a diet with 45 E% fat (Cat# SF04‐001, Specialty Feeds, Glen Forrest, Australia), from 6 to 41 weeks of age. After 23 weeks of high fat feeding, mice were placed in metabolic cages (Promethion, Sable Systems, North Las Vegas, NV, United States). Animals were familiarized to the metabolic cages for 3 days, and indirect calorimetry was performed for 3 days. Oral glucose tolerance testing was performed after 30 weeks of high fat feeding; mice were fasted for 5 h, and gavaged with 2 g glucose per kg lean mass. An insulin tolerance test was performed at 20 weeks after 6 h fasting, by giving an intraperitoneal injection of 3 U/kg lean mass Actrapid human insulin (Novo nordisk, Bagsværd, Denmark). Blood samples were taken from the tip of the tail.

For the wheel running experiment, chow‐fed, female mice were singly housed in cages with either a locked or a functional running wheel (Columbus Instruments, Columbus, OH, USA), from approximately 8 to 14 weeks of age (6 weeks intervention). A subset was sacrificed after 3 weeks.

For treadmill experiments, male, chow‐fed *Dcn* ^−/−^ and *Dcn* ^+/+^ mice were familiarized to a motorized treadmill 5 times on separate days. The animals were then exercised until exhaustion using the following incremental speed program: 5 min on 14, 18, 20, 22, and 24 m/min, 35 min 26 m/min, 10 min 28 m/min, 5 min 30 m/min, and 32 m/min until exhaustion. Exercise testing was done blinded, and mice were defined as exhausted when remaining at the back of the treadmill for >5 s despite gentle encouragement. The mice were sacrificed at 15 weeks of age.

Mouse body composition was measured with a body composition analyzer (EchoMRI™, Houston, TX, USA). At the end of the experiments, animals were sedated by isoflurane inhalation (1 L/minute of 4% isoflurane), blood was collected by cardiac puncture, followed by cervical dislocation. Tissues were snap frozen in liquid nitrogen or processed for histology as described below.

### 2.2 Histology and immunofluorescence staining

Tibialis anterior and gastrocnemius muscles were fixed in 10% phosphate‐buffered formalin, embedded in paraffin, sectioned, and stained with hematoxylin and eosin for routine histology at the Garvan Histopathology Core Facility (Garvan Institute of Medical Research, Darlinghurst, NSW, Australia). Cross sections from whole muscles were scanned with a 5× lens on a Leica DM 6000 Power Mosaic microscope with a stepping stage for mosaic image acquisition. Fiber cross‐sectional area and Feret diameter were measured by manual annotation of 50–150 fibers per section, using ImageJ software (RRID:SCR_003070).

For immunofluorescence staining, quadriceps muscle tissue was embedded in O.C.T.™ Compound (Tissue‐Tek®, Sakura Finetek, Tokyo, Japan) and frozen in isopentane cooled by liquid nitrogen. Tissues were cryosectioned, fixed in 4% paraformaldehyde, incubated in 1% horse serum, then incubated with primary antibodies (Decorin; R and D Systems Cat# AF1060, RRID:AB_2090386) in 1% bovine serum albumin, 1% horse serum, and 0.03% triton X‐100 in PBS overnight. The slides were then incubated with DAPI and a Cy™3 conjugated secondary antibody (Cat# 705‐166‐147, Jackson ImmunoResearch, RRID:AB_2340413). Image acquisition was done on a Leica DM5500 microscope.

### 2.3 Immunoblotting

Muscle tissue was homogenized in RIPA buffer and subjected to SDS‐PAGE with criterion™ TGX™ gels (Bio‐Rad, Hercules, CA, USA). Protein transfer was done using the Trans‐Blot Turbo transfer system and RTA transfer kit (Bio‐Rad, Hercules, CA, USA). For total protein measurements the membranes were either stained with Ponceau S, or visualized with TGX stain‐free technology (Bio‐Rad, Hercules, CA, USA) for fluorescent detection of proteins. The membranes were then blocked in Tris buffered saline containing 0.1% Tween‐20 and 5% BSA, and incubated over night with primary antibodies in 2.5% BSA in TBS‐T. The following antibodies were used: Total OXPHOS rodent antibody cocktail (MitoScience LLC Cat# MS604, RRID:AB_2629281), glycogen synthase (Cell Signaling Technology Cat# 3886, RRID:AB_2116392), phospho‐glycogen synthase (Cell Signaling Technology Cat# 3891, RRID:AB_2116390), Ppargc1a (Santa Cruz Biotechnology Cat# sc‐13067, RRID:AB_2166218), Tnf (Cell Signaling Technology Cat# 3707, RRID:AB_2240625), PI3 Kinase p85 (Millipore Cat# 06‐497, RRID:AB_310141), Stat‐3 (Cell Signaling Technology Cat# 4904, RRID:AB_331269), and phopho‐Stat3 ((Cell Signaling Technology Cat# 9167, RRID:AB_561284)). For detection we used HRP‐conjugated secondary antibodies (Jackson ImmunoResearch Labs Cat# 111‐035‐144/RRID:AB_2307391 and Cat# 115‐035‐146/RRID:AB_2307392), ECL substrate (Cat# 34076, Thermo Scientific, Rockford, IL, USA) and the ChemiDoc™ Touch Imaging System (Bio‐Rad, Hercules, CA, USA). Quantification of band intensities was done in ImageJ.

### 2.4 Gene expression analyses

For reverse transcriptase quantitative PCR (RT‐qPCR), tissues were homogenized in TRI Reagent solution (Thermo Fisher, Waltham, MA, USA) and phase‐separated by adding chloroform. RNA was then isolated with a Nucleospin RNA extraction kit (Cat# 740955, Macherey‐Nagel, Dueren, Germany) according to the manufacturer's instructions. cDNA was synthesized with a Tetro cDNA synthesis kit (Cat# BIO‐65043, Bioline, Meridian Bioscience, Memphis, TN, USA). qPCR was either performed using TaqMan reagents and predeveloped assays from Applied Biosystems (Waltham, MA, USA), or Bio‐Rad SsoAdvanced™ Universal SYBR® Green Supermix on a CFX96 Touch instrument (Bio‐Rad, Hercules, CA, USA). Primer sequences used for the SYBR Green based assay were as follows: *Dcn* (spanning exon 2–3: TCGAGTGGTGCAGTGTTCTG, TAGCAAGGTTGTGTCGGGTG), *Dcn* (spanning exon 7–8: TGAGGGAACTCCACTTGGACA, TGCGGAGATGTTGTTGTTGTG), and *Tbp* (AGCCTTCCACCTTATGCTCAG, GCCGTAAGGCATCATTGGACT). Gene expression was calculated using the 2^−∆∆Ct^ method, with *Tbp* as a reference gene.

### 2.5 Statistics

Statistical testing was done with 2‐way ANOVA followed by Fisher's Least Significant Difference test or repeated measures ANOVA. Simple group differences were tested with Student's *t*‐test or Mann Whitney test. Statistical analyses were done in GraphPad Prism v10 (RRID:SCR_002798).

## 3 RESULTS

### 3.1 Decorin deficiency does not influence obesity or metabolism in high‐fat diet‐fed mice

**FIGURE 1:**
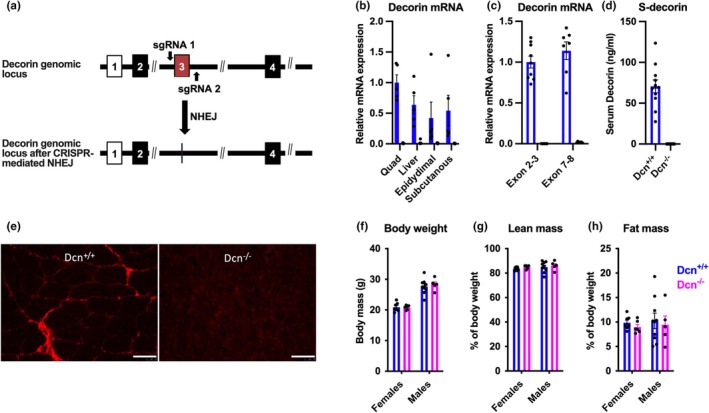
Generation of decorin deficient mice. (a) CRISPR/Cas9 gene targeting strategy to generate decorin deficient mice. (b) Decorin mRNA expression in quadriceps muscle, liver and epidydimal and subcutaneous adipose tissue of male *Dcn* ^+/+^ and *Dcn* ^−/−^ mice (*n* = 5–7). (c) Decorin mRNA expression of transcripts corresponding to exon 2–3 and 7–8 in muscle tibialis anterior of female *Dcn* ^+/+^ and *Dcn* ^−/−^ mice (*n* = 7–9). (d) Serum decorin concentration in *Dcn* ^+/+^ and *Dcn* ^−/−^ male mice (*n* = 12). (e) Representative image from immunofluorescence staining of decorin in quadriceps muscle of *Dcn* ^+/+^ and *Dcn* ^−/−^ mice. Scale bar represents 50 μm. (f–h) Body weight, lean mass, and fat mass in male and female mice fed a chow diet, at 11 weeks of age (*n* = 5–9).

We generated decorin deficient mice (*Dcn* ^−/−^) on a C57BL/6J genetic background using a CRISPR/Cas9 gene targeting strategy, leading to removal of exon 3 and the introduction of an early stop codon due to a shift in the reading frame (Figure 1a). Comparison of Decorin mRNA expression in Dcn^+/+^ and Dcn^−/−^ littermates in skeletal muscle, adipose tissue, and liver revealed high expression in all three tissues in Dcn^+/+^ mice, but no expression in Dcn^−/−^ mice (TaqMan primers, Figure 1b). We next used two sets of primer pairs for quantification of *Dcn* mRNA expression (Figure 1c). The expression of *Dcn* transcripts corresponding to the exon 2 to 3 junction was around the detection limit in Dcn^−/−^ mice (CT_mean_ = 34.9 vs. CT_mean_ = 22.7 in Dcn^+/+^ mice). In contrast, expression was notable but highly reduced in Dcn^−/−^ mice when amplifying across exon 7–8 (CTmean = 27.5 vs. CTmean = 22.1 in Dcn+/+ mice), indicating nonsense‐mediated mRNA decay of the truncated *Dcn* mRNA transcript.

To further validate that decorin protein expression was also knocked out, we measured decorin protein levels in plasma using ELISA. Decorin protein was detected in Dcn^+/+^ mice, while decorin concentrations were below the detection limit in the plasma of all tested Dcn^−/−^ mice (Figure 1d). Furthermore, immunostaining of decorin in quadriceps muscle revealed that decorin was present in the endomysium surrounding individual muscle fibers in Dcn^+/+^ but not in Dcn^−/−^ mice (Figure 1e).

**FIGURE 2:**
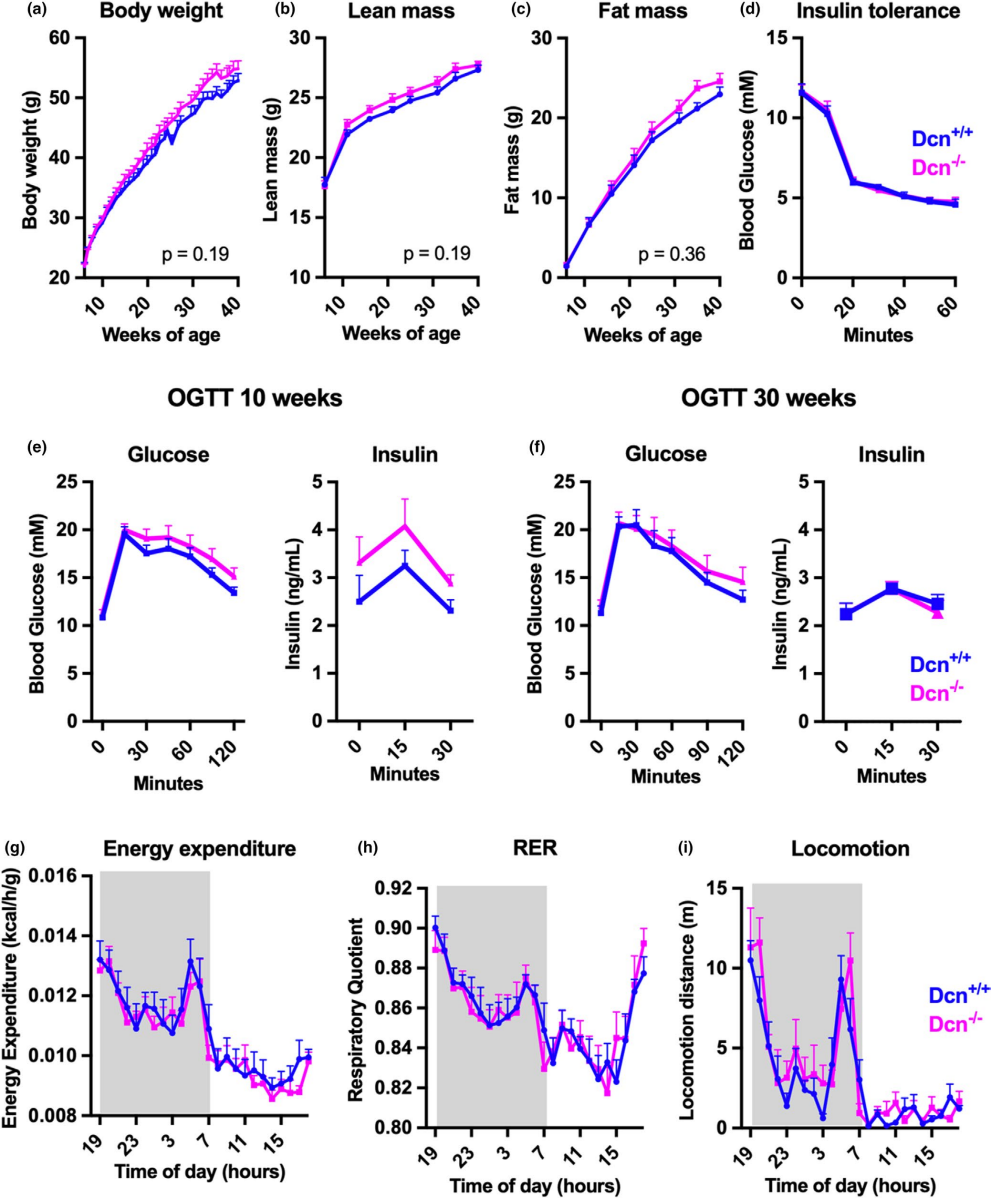
Decorin deficiency does not influence body composition, glucose metabolism, or substrate oxidation in mice on a high fat diet. *Dcn* ^+/+^ mice and *Dcn* ^−/−^ littermates were placed on a HFD for 35 weeks (*n* = 12). Body weight was measured weekly (a), and lean mass (b) and fat mass (c) was measured using MRI‐NMR technology every 4–5 weeks. The effect of an injection of insulin on blood glucose was tested after 20 weeks (d). Oral glucose tolerance testing was performed after 10 (e) and 30 weeks (f). At 23 weeks, metabolic monitoring was performed over 3 days with metabolic cages. Energy expenditure (g), respiratory exchange ratio (h) and locomotion activity (i) on day 2. Shaded area represents the dark phase.

Overall, decorin deficient mice appeared normal upon gross examination, that is, shiny coat, good body condition, alert and active, and absence of signs of illness (i.e., lethargy, hunched posture, and grimace indicative of pain). Furthermore, body weight or body composition were not altered in 11‐week‐old male and female mice fed a chow diet (Figure 1f–h). To study the impact of decorin deficiency on the development of obesity, male mice were fed a high fat diet for 35 weeks. Both *Dcn* ^+/+^ and *Dcn* ^−/−^ littermates developed similar levels of obesity (Figure 2a–c), with a fat mass of around 44% of body weight (range 39%–49%) at the end of the study. Oral glucose tolerance testing at 10 and 30 weeks of age revealed similar glucose tolerance in both groups (Figure 2e,f). Decorin deficient animals also had a similar response to an insulin tolerance test as compared to *Dcn* ^+/+^ (Figure 2d). At 35 weeks of age, the animals were housed in metabolic cages, and we did not observe any genotype differences in energy expenditure, respiratory exchange ratios, or locomotion activity levels (Figure 2g–i).

### 3.2 Decorin deficiency, skeletal muscle, and exercise

Many muscle‐secreted factors execute their function locally in skeletal muscle (Piccirillo, 2019). Due to the role of decorin as a matrix proteoglycan, we further explored the potential roles of decorin in skeletal muscle. Analysis of quadriceps muscle from high‐fat fed mice showed that decorin deficiency did not influence mRNA expression of metabolic marker genes (*Ppargc1a*, *Cs*, *Tfam*, *G6pc3*) or the protein expression of mitochondrial oxidative phosphorylation complexes, glycogen synthase and phospho‐glycogen synthase, Ppargc1a, Tnf, PI3 Kinase p85, Stat‐3, and phospho‐Stat3 (Figure S1).

**FIGURE 3:**
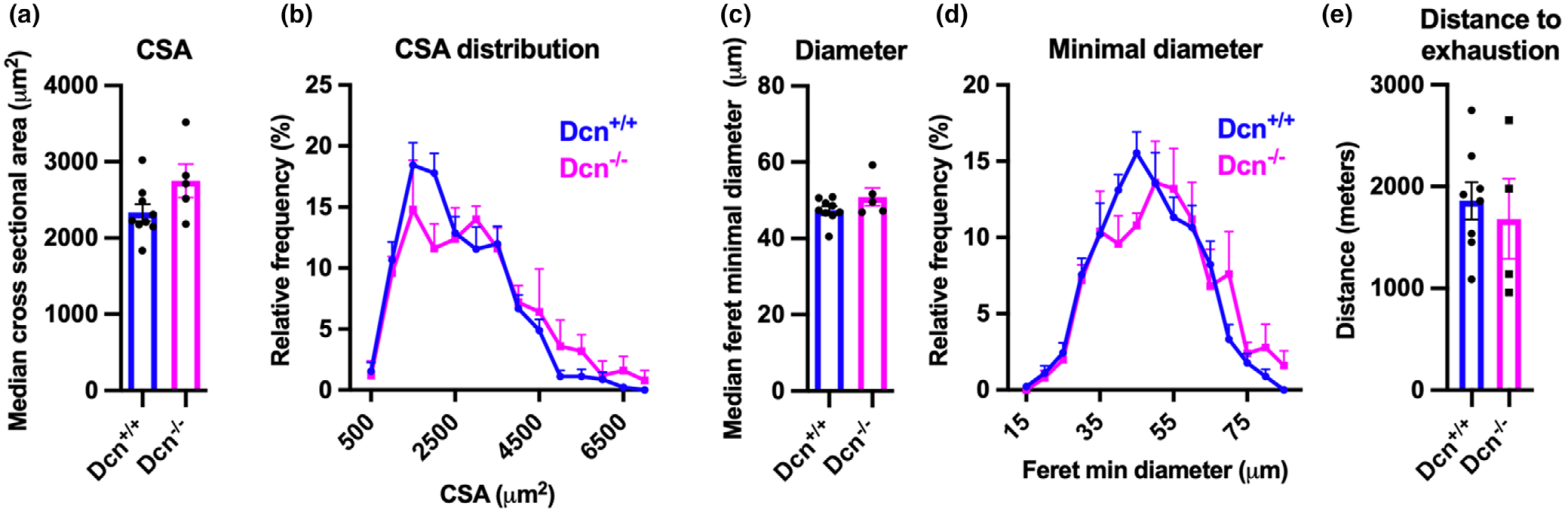
The effect of decorin deficiency on skeletal muscle and exercise capacity. Male mice *Dcn* ^+/+^ and *Dcn* ^−/−^ were fed a chow diet until 14–15 weeks of age. (a–d) Muscle fiber size of muscle tibialis anterior (*n* = 5–9). Median cross‐sectional area (CSA) and CSA distribution (a, b) and Ferets minimum diameter (c, d). (e) Aerobic running capacity of male mice was tested with a graded treadmill running program (*n* = 4–8) and shown as distance to exhaustion. Bars represent mean + SEM.

To study the potential effects of decorin deficiency on acute and long‐term training, we utilized chow fed male and female mice, respectively. *Dcn* ^−/−^ mice displayed normal muscle morphology as analyzed by H&E staining of sections from the tibialis anterior muscle (Figure S2). The size distribution of these muscle fibers, measured as cross‐sectional area and minimum Feret diameter, was similar in *Dcn* ^+/+^ and *Dcn* ^−/−^ mice (Figure 3a–d).

To investigate the effect of decorin deficiency on aerobic exercise capacity, male chow‐fed mice were exercised until fatigue on a motorized treadmill. *Dcn* ^−/−^ mice ran an equal distance to *Dcn* ^+/+^ mice, indicating similar capacities for endurance exercise (Figure 3e).

**FIGURE 4:**
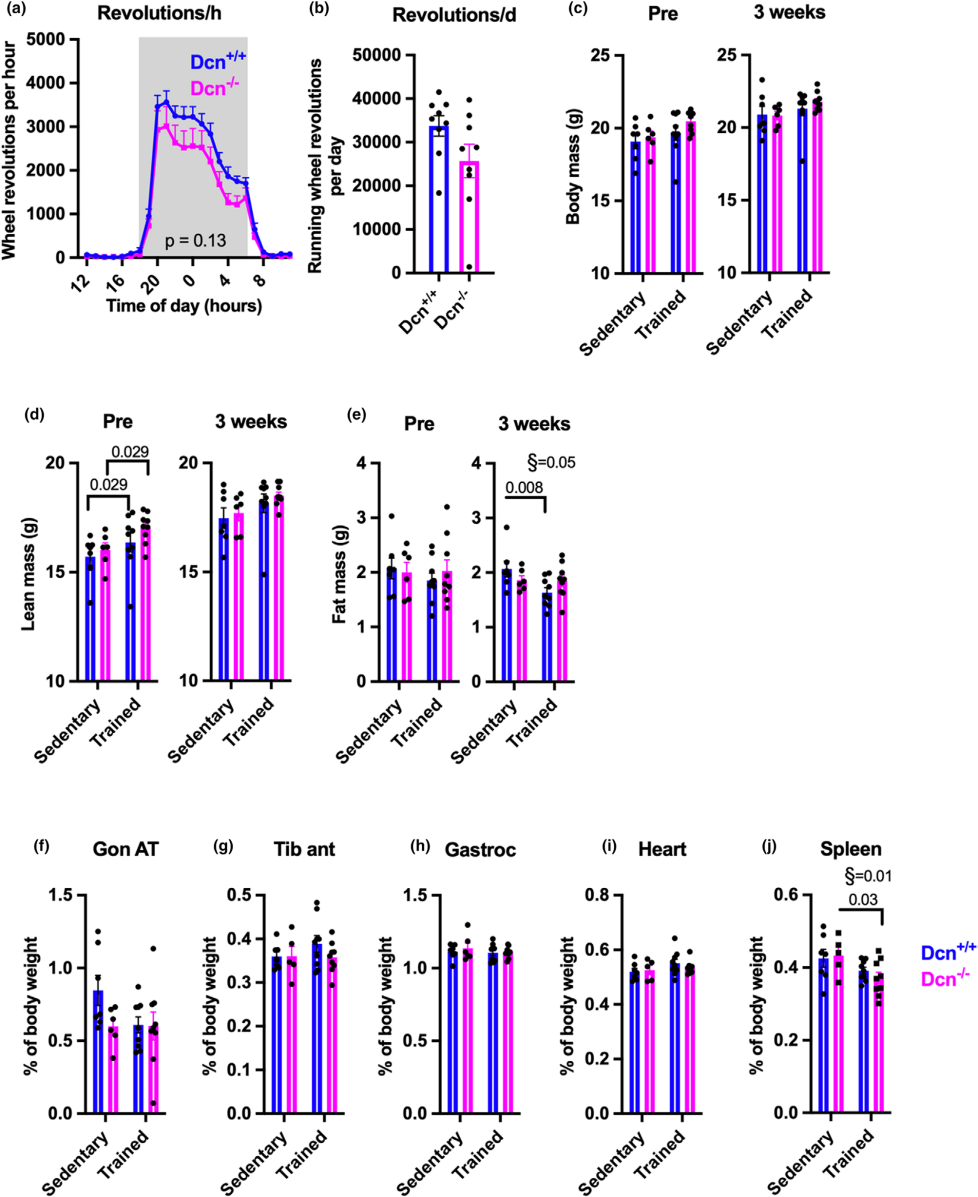
The effect of decorin deficiency on exercise adaptation. Female *Dcn* ^*+/+*^ and *Dcn* ^*−/−*^ were singly housed in home cages with access to locked (Sedentary) or functional (Trained) running wheels for 6 weeks. Voluntary running wheel activity is shown as running wheel revolutions or per hour (a) or per day (b). Shaded area in (a) represent dark phase. (c–e) Body weight, lean mass, and fat mass at baseline (approximately 8 weeks of age) and after 3 weeks of wheel running (*n* = 6–9). (f–i) Tissue weights at after 6 weeks of wheel running (*n* = 6–9); gonadal adipose tissue (f), tibialis anterior muscle (g), gastrocnemius muscle (h), heart (i), and spleen (j). Bars represent mean + SEM. Statistical testing was done with 2‐way ANOVA, with § indicating overall training effect, and brackets indicating significant differences with *p* values from a Fishers Least Significant Difference post hoc test.

To determine the effects on long‐term, voluntary exercise, female mice were single housed in cages equipped with either locked or functional running wheels for 6 weeks. Both *Dcn* ^−/−^ and *Dcn* ^+/+^ mice had a peak in running activity between 7 pm and 7 am (Figure 4a). There was a tendency for reduced daily running in Dcn^−/−^ mice; on average, daily running distance was 33,756 and 25,694 revolutions/d in *Dcn* ^+/+^ and *Dcn* ^−/−^ mice, respectively (*p* = 0.09, Figure 4b). Of note, there were no differences in ambulatory activity in obese Dcn^−/−^ mice as compared to Dcn^+/+^ mice (Figure 2i). To determine the effect of voluntary exercise on body composition, the mice were subjected to NMR‐MRI screening at baseline (pre) and after 3 weeks of wheel running (Figure 4c–e). *Dcn* ^+/+^ mice exposed to wheel running had a lower fat mass compared to sedentary mice (*p* = 0.008, Figure 4e), but the same was not observed in *Dcn* ^−/−^ mice. This genotype difference was further reflected in a tendency for larger gonadal adipose depots in sedentary *Dcn* ^+/+^ mice at the end of the intervention (Figure 4f). We did not observe any significant mass differences in tibialis anterior muscle, gastrocnemius muscle, or heart, while spleen mass was significantly lower with wheel running (Figure 4g–j).

**FIGURE 5:**
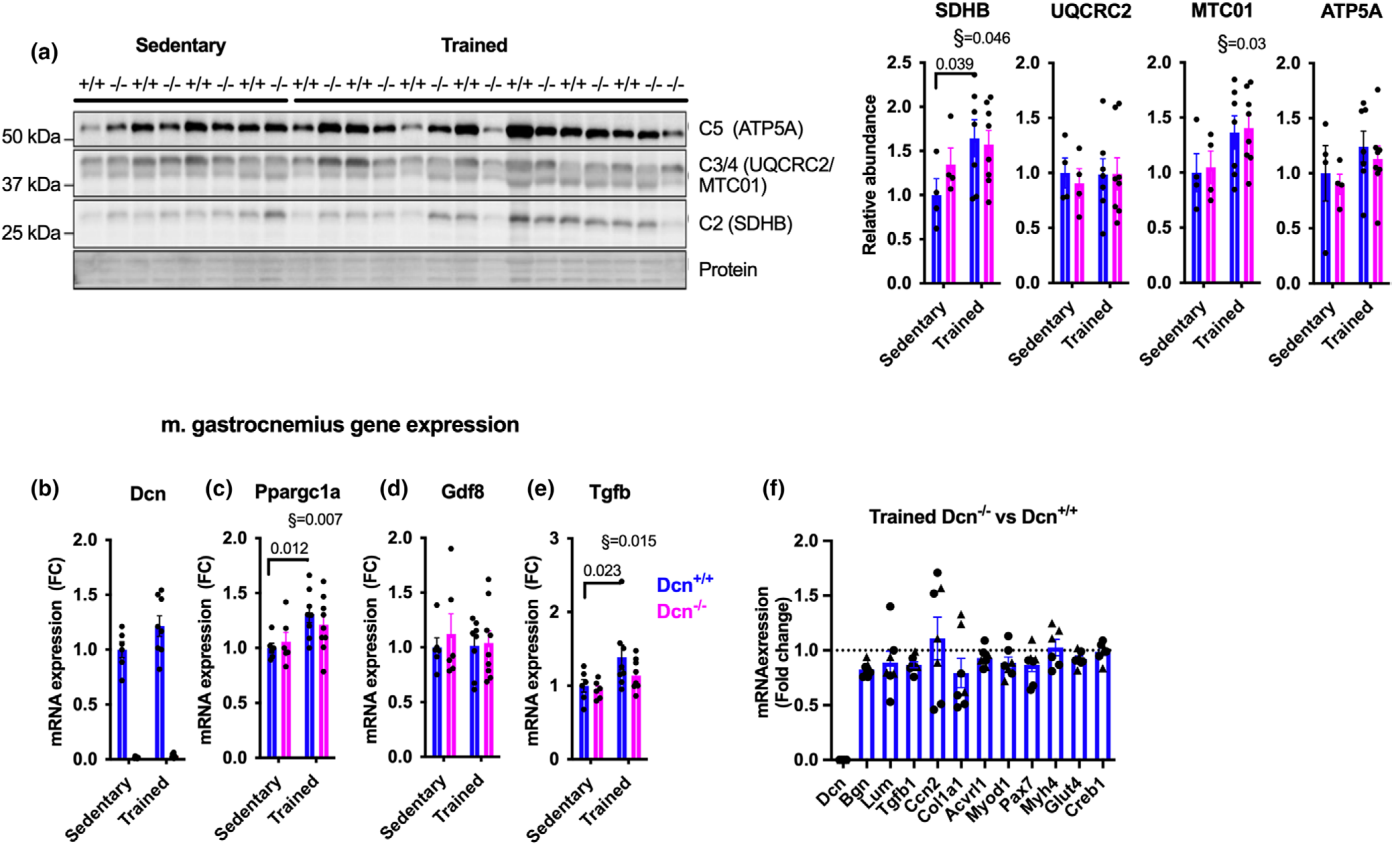
The effect of decorin deficiency on exercise adaptation in female mice. Female *Dcn* ^+/+^ and *Dcn* ^−/−^ were singly housed in home cages with access to locked (Sedentary) or functional (Trained) running wheels. (a) Western blotting of oxidative phosphorylation proteins in muscle tibialis anterior of sedentary and trained mice after 6 weeks of intervention (*n* = 4–8); SDHB (complex II), UQCRC2 (complex III), MTC01 (complex IV), and ATP5A (complex V). Quantified and expressed relative to the sedentary *Dcn* ^+/+^ group. (b–e) mRNA expression in gastrocnemius muscle (6 weeks training, *n* = 6–9), presented relative to the sedentary *Dcn* ^+/+^ group. (f) mRNA expression in tibialis anterior muscle, *Dcn* ^−/−^ trained relative to the *Dcn* ^+/+^ trained mice, 3 (triangles) or 6 weeks (circles) training. Bars represent mean + SEM. § Statistical testing was done with 2‐way ANOVA, with § indicating overall training effect, and brackets indicating significant differences with *p* values from a Fishers Least Significant Difference post hoc test.

To study metabolic adaptation of skeletal muscle, we measured the abundance of mitochondrial proteins and the mRNA expression of exercise‐responsive genes (Figure 5). The abundance of some mitochondrial oxidative phosphorylation proteins (SDHB and MTC01) was generally increased in response to training (Figure 5a), indicating mitochondrial adaptation. Furthermore, gene expression analyses in gastrocnemius muscle revealed increased expression of the metabolic regulator PGC1α (*Ppargc1a*) in response to exercise (*p* = 0.007 by ANOVA, Figure 5c). In post hoc analyses, these effects of exercise reached statistical significance in the *Dcn* ^+/+^ group, but not in the *Dcn* ^−/−^ group, suggesting that long‐term exercise may induce a more pronounced adaptation in *Dcn* ^+/+^ mice as compared to *Dcn* ^−/−^ mice. However, we did not observe any statistically significant differences between trained *Dcn* ^−/−^ and trained *Dcn* ^+/+^ mice.

Decorin has been identified as a negative regulator of myostatin (*Gdf8*) and TGFβ. Myostatin mRNA was not regulated by exercise or decorin deficiency (Figure 5d). However, *Tgfb*, which is known to increase after long‐term exercise, was upregulated in trained muscle, but only in *Dcn* ^+/+^ mice (Figure 5e). Because altered TGFβ activity may influence muscle development, we investigated the expression of TGFβ target genes and myogenic genes in trained *Dcn* ^+/+^ and *Dcn* ^−/−^ mice. Decorin deficient mice had a normal expression of *Tgfb*, *Ccn2, Col1a1*, and *Acvrl1*, and myogenic factors such as *Pax7* and *Myod1* (Figure 5f), as compared to *Dcn* ^+/+^ mice. Lastly, we hypothesized that decorin deficiency gives a compensatory increase in the mRNA expression of closely related proteoglycans. However, lumican or biglycan (Figure 5f), which belong to the same family of small leucine‐rich proteoglycans and share structural similarity to decorin, were not differentially expressed in *Dcn* ^−/−^ mice.

## 4 DISCUSSION

We developed a new decorin deficient mouse model to investigate the effects of decorin deficiency on adiposity, glucose tolerance, and aerobic exercise capacity. Overall, decorin deficient mice appeared normal upon gross examination, consistent with observations from other studies (Danielson et al., 1997; Mellgren et al., 2015). The first decorin knockout mouse model was generated by Danielson et al. (1997). Decorin deficient mice had abnormal collagen fibril structure in skin and tendon, which was accompanied by thin and fragile skin. Otherwise, they did not display any gross anatomical abnormalities or behavioral differences; they had normal growth and fertility, and normal levels of standard hematological and blood chemistry markers. A decorin deficient mouse model has also been phenotyped by the International Mouse Phenotyping Consortium, revealing no impact on the physiological systems tested (https://www.mousephenotype.org/data/genes/MGI:94872) (Dickinson et al., 2016; International Mouse Phenotyping Concortium, 2025). Later studies have further characterized tendon in decorin deficient mice, with results indicating ultrastructural changes in collagen (Dourte et al., 2012), but with mixed effects on elastic or compressive properties (Dourte et al., 2012; Robinson et al., 2005). Decorin deficiency impacts wound healing (Järveläinen et al., 2006), cornea injury (Schönherr et al., 2004), cancer development (Bi et al., 2008), or diabetic kidney (Merline et al., 2009; Williams et al., 2007). In the heart, lack of decorin did not influence fibrosis in healthy myocardium (despite some effect on collagen ultrastructure), but influenced scar formation after myocardial infarction (Weis et al., 2005). Decorin deficiency therefore appears to exert more significant effects during stress or injury compared to under basal conditions, likely due to compensatory mechanisms.

Little is known regarding the effect of decorin on obesity or insulin resistance. Previous studies have reported a higher expression of decorin in adipose tissue of obese and diabetic patients (Pessentheiner et al., 2020). In a study by Bolton et al. (2008), plasma decorin was 12% higher in people with type 2 diabetes compared to healthy controls. However, decorin mRNA expression in adipose tissue is also increased after weight loss induced by bariatric surgery (Svärd et al., 2019). In a previous study by Svärd et al. (2019), high‐fat diet‐fed mice with a truncated *Dcn* gene had reduced glucose tolerance compared to wild type mice. The decorin deficient mice also had increased feed efficiency, meaning that they gained less weight relative to their food intake. These results are contradictory to the findings in our *Dcn* ^−/−^ model, as we did not observe any effects of decorin deficiency on oral glucose tolerance after 10 and 30 weeks on a high‐fat diet. Furthermore, there were no differences in insulin tolerance, body weight and body composition, activity levels, or substrate oxidation rates.

Decorin is the most abundant proteoglycan in skeletal muscle (Kritikaki et al., 2021), with potential effects on muscle development, repair, and adaptation via its ability to modulate multiple signaling pathways and ECM organization (Brandan & Gutierrez, 2013a, 2013b; Buraschi et al., 2019; Järveläinen et al., 2015; Pessentheiner et al., 2020). Several in vitro studies have demonstrated that decorin can increase myogenesis via altered myostatin or TGFβ signaling (Brandan & Gutierrez, 2013a; Kishioka et al., 2008; Li et al., 2007; Suzuki et al., 2013). Kanzleiter et al. overexpressed decorin in the skeletal muscle of mice and measured a slight increase in the mRNA expression of myostatin and some myogenic genes (*Myod1*, follistatin, and akirin1) and a 30% reduction in atrophy‐related genes (atrogin 1 and murf1) (Kanzleiter et al., 2014). Although these results may indicate a role for decorin in muscle development or hypertrophy, physiological evidence is lacking. In our study, we did not observe any major effects of decorin deficiency on murine skeletal muscle, including muscle morphology, expression of metabolic and mitochondrial markers, myogenic factors, or TGFβ responsive genes. However, there was a tendency for reduced voluntary running wheel activity in decorin‐deficient mice. Furthermore, trained Dcn^+/+^ mice had a lower fat mass than Dcn^−/−^ mice, and Dcn^+/+^ mice seemed to have a more pronounced response in exercise‐responsive muscle markers such as *Ppargc1a*, possibly due to differences in physical activity levels.

The absence of major effects of decorin deficiency in our study may reflect compensatory mechanisms, and potential phenotypes may be more evident in models of greater homeostatic stress, such as diabetes or muscle injury. Decorin has been implicated in muscle regeneration following injury (Li et al., 2007), suggesting potential roles in eccentric exercise or muscle strain. Its involvement in resistance exercise adaptations, such as muscle hypertrophy, remains unexplored. The lack of effects may also be explained by the context‐dependent nature of decorin. For instance, decorin inhibits TGFβ signaling, but is also required for TGFβ signaling in proliferating myoblasts (Brandan & Gutierrez, 2013a; Cabello‐Verrugio & Brandan, 2007). Furthermore, decorin modulates the activity of a multitude of ligands and receptors, which makes it challenging to study the biological effects. Additionally, type 2 errors cannot be ruled out, particularly in the long‐term exercise experiments, where Dcn^−/−^ mice showed a trend toward reduced running wheel activity.

## 5 CONCLUSION

In conclusion, the absence of decorin did not influence the development of obesity or glucose intolerance and had no major effects on skeletal muscle fiber size or expression of genes related to metabolism, myogenesis, or ECM formation.

## AUTHOR CONTRIBUTIONS

Conceived and designed research: CLE, MP, MW, MAF, and MH. Received funding: MAF and KTD. Performed experiments: CLE, AD, and SLF. Analyzed data: CLE and MH. Interpreted results of experiments: MH and CLE. Prepared figures and drafted manuscript: MH. All authors edited and revised the manuscript and approved the final version of the manuscript.

## FUNDING INFORMATION

Research was supported by project grants from the National Health and Medical Research Council Australia (APP1042465, APP1041760, recipient: MAF) and The Throne Holst Foundation (recipient: KTD).

## CONFLICT OF INTEREST STATEMENT

MAF is a shareholder and scientific advisor for N‐Gene Pharmaceuticals; MAF is the founder and shareholder of Celesta Therapeutics. The authors declare that they have no other competing interests.

## ETHICS STATEMENT

All animal experiments were conducted in strict accordance with the Australian Code for the Care and Use of Animals for Scientific Purposes. The study protocol was reviewed and approved by the Alfred Medical Research Education Precinct or Garvan Institute/St Vincent's Animal Ethics Committee. All procedures were designed to minimize animal suffering and reduce the number of animals used, adhering to the principles of the 3Rs (Replacement, Reduction, and Refinement). Mice were housed in a controlled environment with appropriate temperature, humidity, and light cycles, and were provided with ad libitum access to food and water. All personnel involved in the study were trained in proper animal handling and care to ensure humane treatment throughout the experimental process.

## Supporting information


Figure S1.

Figure S2.

## Data Availability

The data that support the findings of this study are available on request from the corresponding author (MH).
