# Supplementary material for: The proteoglycan decorin does not influence adiposity, glucose tolerance, or aerobic exercise capacity in mice
Source: Physiol Rep. 2025 Jul 4;13(13):e70424. doi: 10.14814/phy2.70424 (PMC12227656; doi:10.14814/phy2.70424)
Supplement: Supplementary file 1 — Figure S1. [file PHY2-13-e70424-s001.zip › Supplementary Figure 1 caption.docx]

**Supplementary Figure 1.** *Dcn^+/+^* mice and *Dcn^-/-^* littermates were placed on a HFD for 35 weeks (n=12). A; mRNA expression in quadriceps muscle. B; Protein expression in gastrocnemius muscle measured with western blotting. Band intensities were calculated relative to loading control (ponceau stain or TGX stain-free fluorescent detection of proteins). A total OXPHOS antibody cocktail was used for detection of mitochondrial oxidative phosphorylation complexes, and the band detected slightly above 50 kDa was assumed to be complex 5 (ATP5A), complex 4 (MTC01) was assumed to be the band at around 40 kDa and complex 2 (SDHB) was detected at around 30 kDa. Data are presented as fold change, *Dcn^-/-^* relative to *Dcn^+/+^.* Statistical testing was done with a student one-sample t-test. *p<0.05, significant difference from 1.
