# Supplementary figures and images for: The proteoglycan decorin does not influence adiposity, glucose tolerance, or aerobic exercise capacity in mice
Source: Physiol Rep. 2025 Jul 4;13(13):e70424. doi: 10.14814/phy2.70424 (PMC12227656; doi:10.14814/phy2.70424)

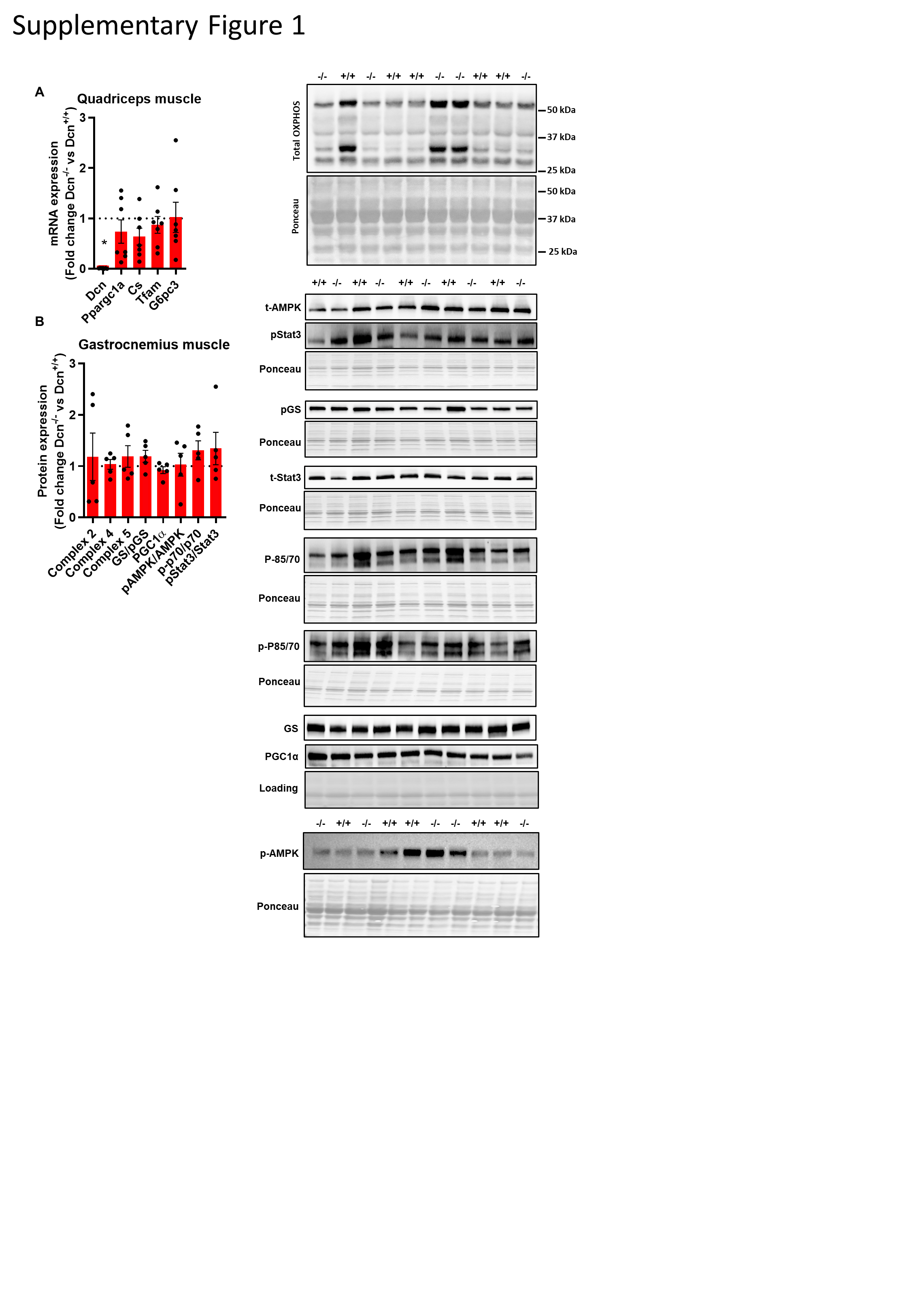

Supplement: Supplementary file 1 — Figure S1. [file PHY2-13-e70424-s001.zip › SFig1.tif]

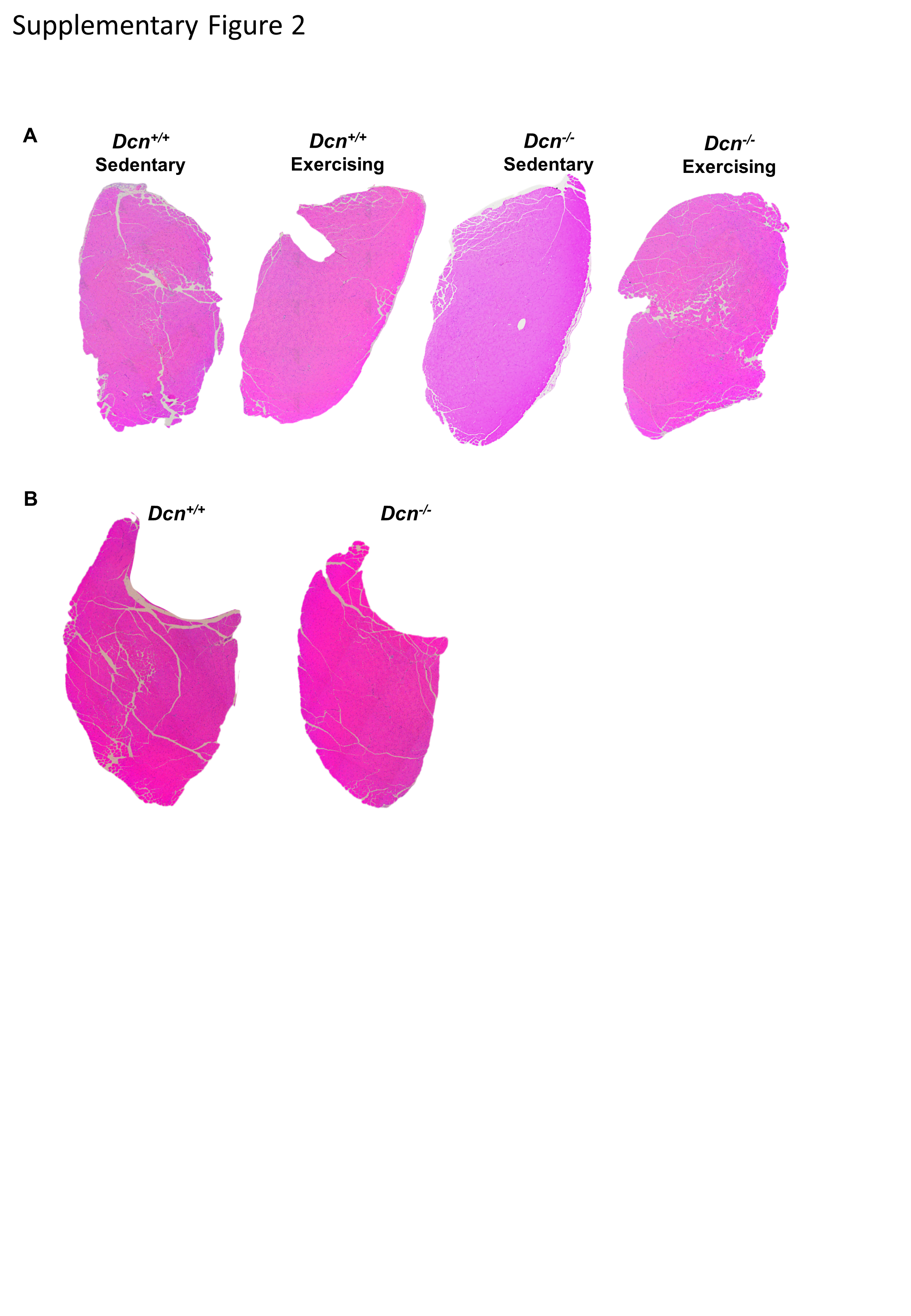

Supplement: Supplementary file 2 — Figure S2. [file PHY2-13-e70424-s002.zip › SFig2.tif]
