# Supplementary material for: The proteoglycan decorin does not influence adiposity, glucose tolerance, or aerobic exercise capacity in mice
Source: Physiol Rep. 2025 Jul 4;13(13):e70424. doi: 10.14814/phy2.70424 (PMC12227656; doi:10.14814/phy2.70424)
Supplement: Supplementary file 2 — Figure S2. [file PHY2-13-e70424-s002.zip › Supplementary Figure 2.docx]

**Supplementary Figure 2.** Cross sectional view of the tibialis anterior muscle from female (A) and male (B) *Dcn^+/+^* and *Dcn^-/-^* mice. Stained with Hematoxylin and eosin stain.
